# Effects of PARP Inhibitors on Subsequent Platinum-Based Chemotherapy in Patients with Recurrent Ovarian Cancer

**DOI:** 10.3390/cancers16152651

**Published:** 2024-07-25

**Authors:** Tetsuya Kokabu, Yosuke Tarumi, Kota Aoki, Ayaka Okamura, Kohei Aoyama, Hisashi Kataoka, Kaori Yoriki, Taisuke Mori

**Affiliations:** Department of Obstetrics and Gynecology, Graduate School of Medical Science, Kyoto Prefectural University of Medicine, Kyoto 602-8566, Japan; y-tarumi@koto.kpu-m.ac.jp (Y.T.); k-aoki@koto.kpu-m.ac.jp (K.A.); aokamura@koto.kpu-m.ac.jp (A.O.); k-aoyama@koto.kpu-m.ac.jp (K.A.); hlm-11@koto.kpu-m.ac.jp (H.K.); kaorix26@koto.kpu-m.ac.jp (K.Y.); moriman@koto.kpu-m.ac.jp (T.M.)

**Keywords:** ovarian cancer, PARP inhibitor, bevacizumab, platinum free interval

## Abstract

**Simple Summary:**

Poly(adenosine diphosphate–ribose) polymerase inhibitors (PARP-is) play crucial roles in the treatment of ovarian cancer. However, the best therapeutic strategy for recurrence during PARP-i maintenance therapy remains unknown. The aim of this study was to elucidate the efficacy of platinum-based chemotherapy after PARP-i treatment in recurrent ovarian cancer. Eighteen patients were enrolled in the present study. The median progression-free survival (PFS) for all patients was 6.5 months, and the median overall survival (OS) was 17.6 months. The evaluation of the risk factors for PFS revealed that bevacizumab use in subsequent therapies was significantly associated with extended PFS. The median PFS was significantly longer in the chemotherapy with the bevacizumab group than in chemotherapy alone (8.9 months vs. 3.1 months, log-rank *p* = 0.022). The present study highlighted that platinum-based chemotherapy with bevacizumab in subsequent therapies would provide substantial benefits in the PFS of patients with recurrent ovarian cancer.

**Abstract:**

The clinical outcomes in patients with ovarian cancer have been significantly improved by Poly(adenosine diphosphate–ribose) polymerase inhibitors (PARP-is). However, the best therapeutic strategy for recurrence during PARP-i maintenance therapy remains unknown. Herein, we elucidated the efficacy of platinum-based chemotherapy after PARP-i treatment in recurrent ovarian cancer. Eligible patients had experienced relapses during PARP-i maintenance therapy lasting at least 6 months and had received subsequent platinum-based chemotherapy at our institution between January 2019 and March 2024. Progression-free survival (PFS), overall survival (OS), and risk factors for PFS were evaluated. Sixty-six patients were assessed for eligibility and eighteen were enrolled. The median follow-up period was 14.5 months. The PFS and OS of all patients were 6.5 and 17.6 months, respectively. The evaluation of the risk factors for PFS revealed that age, pathological type, duration of PARP-i maintenance therapy, prior lines of chemotherapy, and PARP-i dose reduction were not significant prognostic markers. However, bevacizumab use in subsequent therapies significantly extended the PFS. The median PFS was 3.1 months in the chemotherapy-alone group and 8.9 months in the chemotherapy with bevacizumab group (log-rank *p* = 0.022). Platinum-based chemotherapy with bevacizumab in subsequent therapies would provide substantial benefits in the PFS of patients with recurrent ovarian cancer.

## 1. Introduction

Ovarian, fallopian tube and primary peritoneal cancers have poor prognosis, with relapse occurring in more than 80% of cases [1]. Multidisciplinary therapy combining cytotoxic anticancer agents with anti-VEGF antibodies and extended surgery has traditionally been performed in patients with these cancers [2,3,4]. In recent years, the advent of poly adenosine diphosphate–ribose polymerase inhibitors (PARP-is), such as olaparib and niraparib, has dramatically improved their oncologic outcomes [1,5,6,7,8,9]. PARP-is exert their effect by trapping PARP on the DNA strand and inducing replication stress that arrests the replication fork, resulting in an increase in DNA damage accumulation. Consequently, trapped PARP can lead to replication fork collapse and finally cell death, particularly in cells with homologous recombination (HR) deficiency [10]. Although a response to platinum-based agents does not ensure a response to PARP-is, sensitivity to platinum-based agents is considered a crucial clinical biomarker for predicting the response to PARP-is in these cancers [11]. Unfortunately, a substantial proportion of patients experience disease progression during maintenance therapy with PARP-is. The restoration of HR pathway function, which results in an acquired resistance to PARP-is and platinum-based agents, is the most well-known and common mechanism [12]. Previous reports have shown that the duration of PARP-i and treatment-free intervals could be predictive markers for platinum agents in subsequent therapies [13,14]. However, the most effective treatment for these patients remains unclear. In the present study, we evaluated the effects of PARP-is on subsequent chemotherapy in patients with recurrent ovarian, fallopian tube, or primary peritoneal cancers.

## 2. Methods

### 2.1. Patients

This retrospective study was approved by the Institutional Review Board of the Kyoto Prefectural University of Medicine (ERB-C-3157). Patients were enrolled if they were 18 years of age or older and received a PARP-i in their most recent regimen for ovarian, fallopian tube, or primary peritoneal cancer. Pathological findings were classified based on the 4th edition of the WHO Classification of Female Reproductive Organs. The enrolled patients experienced malignancy recurrence during PARP-i maintenance therapy that continued for at least 6 months, and had also received subsequent platinum-based chemotherapy at our institution between January 2019 and March 2024. All patients who met the criteria during the experimental period were enrolled in the present study. The regimen of subsequent therapy was selected based on the clinical evidence, adverse events in prior treatments, and the patient’s requests. Bevacizumab administration was determined by the patient’s or physician’s choice. Deleterious germline or somatic BRCA1/2 mutation and HR status were detected with the use of the BRACAnalysis (Myriad, Salt Lake City, UT, USA) and myChoice (Myriad) tests, respectively. All genomic tests were conducted in accordance with Japanese health insurance coverage. Subsequent therapy was selected at the discretion of the physician.

### 2.2. Follow-Up

Gynecological oncologists clinically examined these patients every 1–2 weeks during chemotherapy and 1–3 months after treatment disruption. Medical interviews, physical examinations, and laboratory tests were performed to assess disease status and complications. The follow-up intervals were determined in accordance with the guidelines for treatment of ovarian cancer, fallopian tube cancer, and primary peritoneal cancer of the Japan Society of Gynecologic Oncology. Abdominal computed tomography (CT) was performed every 3 and 6 months or earlier in patients with suspected recurrence. The efficacy of subsequent therapy was evaluated using the Response Evaluation Criteria in Solid Tumors (RECIST) guidelines version 1.1 [15] or cancer antigen 125 (CA-125) response. The response criteria were as follows: complete response (CR), disappearance of all target lesions; partial response (PR), a greater than 30% decrease in the sum of diameters of target lesions; progressive disease (PD), a greater than 20% increase in the sum of diameters of target lesions; stable disease (SD), a lesion not classified as PD or PR. If CA-125 increased more than 2–3-fold, the upper limit of normal, the imaging examination was performed earlier than scheduled. To evaluate treatment safety, adverse events were classified according to the National Cancer Institute Common Terminology Criteria for Adverse Events (CTCAE) version 5.0 after the initiation of treatment.

### 2.3. Statistical Analysis

The Mann–Whitney U test and Student’s *t*-test were utilized in the statistical comparison of risk factors. The definition of progression-free survival (PFS) was the time period between the first day of subsequent therapy and the onset of tumor regrowth and recurrence. The definition of overall survival (OS) was the time period between the confirmed day of recurrence during PARP-i treatment and death. Survival curves were calculated and compared using the Kaplan–Meier method and the log-rank test. Statistical analyses were performed using SPSS Statistics version 27 (IBM, Armonk, NY, USA).

## 3. Results

### 3.1. Patient Characteristics

**Figure 1:**
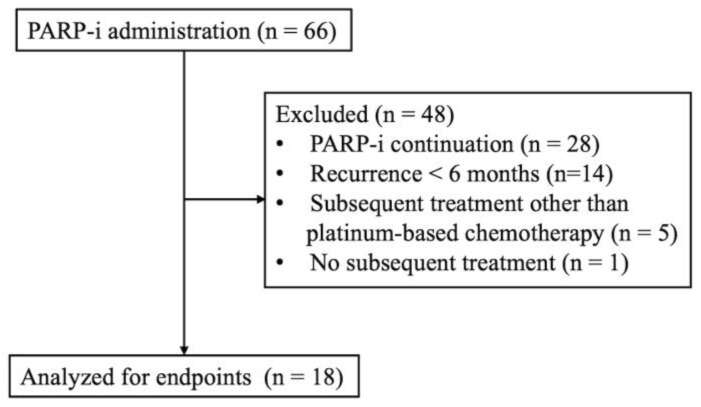
Flowchart of patients’ selection.

The disposition of the participants with detailed information on the excluded patients is shown in Figure 1. Sixty-six patients received PARP-i treatment at our hospital. Among them, data on 18 who experienced relapse during maintenance therapy with a PARP-i after 6 months or more and then received subsequent platinum-based chemotherapy were analyzed. Table 1 shows the baseline characteristics in the present study. The median age was 67.0 (range: 30–80) years, and the median duration of follow-up was 14.5 months. The performance status was 0 in 83.3% of patients. Twelve patients (66.7%) had ovarian cancer, one (5.6%) had fallopian-tube cancer, and five (27.8%) had primary peritoneal cancer. Eighteen patients were included in the analysis, including fifteen (83.3%) patients on olaparib and three (16.7%) on niraparib. The median duration of PARP-i therapy was 9.3 (range: 6.4–18.6) months, and 16 (88.9%) patients continued PARP-i for less than 12 months. Three patients (16.7%) had received prior bevacizumab treatment. Germline or somatic BRCA status was evaluated in six patients, and only one patient had a BRCA mutation.

### 3.2. Efficacy of Subsequent Therapy

**Figure 2:**
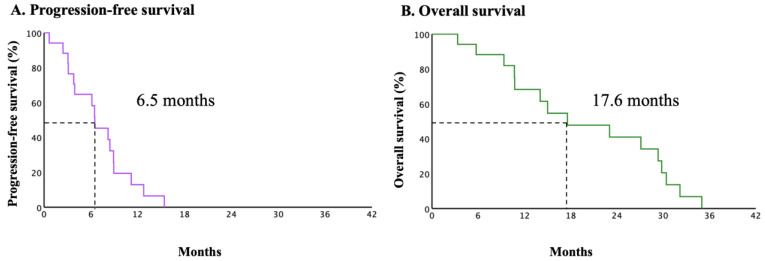
Kaplan–Meier estimates of progression–free survival (**A**) and overall survival (**B**) in overall patients (n = 18).

**Figure 3:**
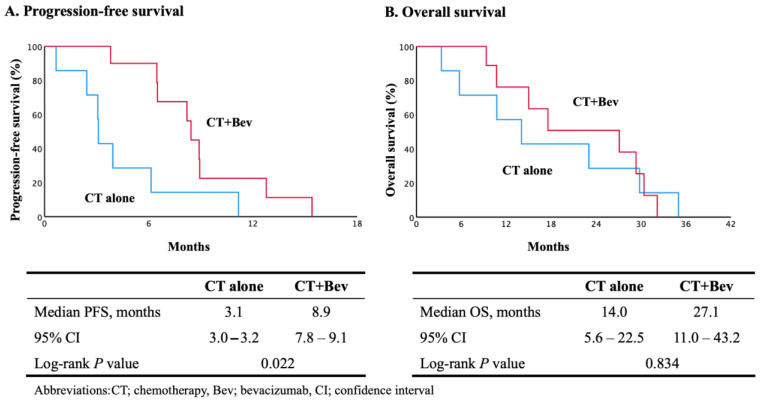
Kaplan–Meier estimates of progression–free survival (**A**) and overall survival (**B**) in subgroup analysis.

Figure 2 shows the oncological outcomes of all patients in the present study. The median PFS and OS were 6.5 (95% CI, 5.7 to 7.9) and 17.6 (95% CI, 6.5 to 28.7) months, respectively. The univariate subgroup analyses of PFS in prognostically relevant patients were performed to evaluate the effects of a PARP-i on subsequent therapy (Table 2). The median scores in Table 1 were used as the numerical cutoffs for each factor. Although bevacizumab use in subsequent therapies was an independent prognostic factor for PFS, the statistical significance of the other factors was not elucidated. The regimen and efficacy of the subsequent therapies are shown in Table 3. Two patients received nedaplatin due to a serious hypersensitive reaction to the prior administration of carboplatin. Fourteen patients received agents other than paclitaxel to avoid suffering from peripheral neuropathy or alopecia. The clinical response rates to platinum-based chemotherapy without and with bevacizumab were 12.5% and 80.0%, respectively. Figure 3 shows the subgroup analyses of PFS and OS with and without bevacizumab. The median PFS was 3.1 months (95% CI, 3.0–3.2) in the chemotherapy-alone group (CT group) and 8.9 months (95% CI, 7.8–9.1) in the chemotherapy with bevacizumab group (CT + Bev group) (log-rank *p* = 0.022). The median OS was 14.0 months (95% CI, 5.6–22.5) in the chemotherapy-alone group and 27.1 months (95% CI, 11.0–43.2) in the chemotherapy with bevacizumab group (log-rank *p* = 0.834).

### 3.3. Adverse Events

Adverse events ≥ grade 2 observed in the present study are shown in Table 4. The most common grade 3 or 4 adverse events were hematological toxicities. G3 or G4 neutropenia, anemia, and thrombocytopenia in the CT group occurred in three (37.5%) patients, three (37.5%) patients, and one (12.5%) patient, respectively. In the CT + Bev group, G3 or G4 neutropenia, anemia, and thrombocytopenia occurred in seven (70.0%) patients, three (30.0%) patients, and five (50.0%) patients, respectively. One patient (10.0%) in the CT + Bev group developed proteinuria (≥G3). Hypertension (≥G2) was observed in three (30.0%) patients in the CT + Bev group, but not in the CT group. None of the patients developed perforations, fistulas, thromboembolic events, cardiac disorders, or treatment-related deaths. Although grade 3 or 4 hematological toxicities were frequently observed in the CT + Bev group, there were no significant differences between the CT and CT + Bev groups (*p* = 0.173).

## 4. Discussion

We demonstrated that relapsed tumors during maintenance therapy with a PARP-i present as platinum-resistant cancers, regardless of the duration of maintenance therapy. Additionally, bevacizumab plays a crucial role in prolonging PFS in subsequent platinum-based chemotherapy for patients with recurrence after PARP-i maintenance therapy.

PARP-is trap PARP on DNA at sites of single-strand breaks (SSBs), hindering repair and promoting their conversion to double-strand breaks in HR-deficient cancers like BRCA1/2-mutant and RAD51-mutant tumors, inducing apoptosis [11,16]. Therefore, PARP-is induce apoptosis in cancer cells through the accumulation of DNA damage. However, PARP-i resistance occurs frequently. The restoration of HR repair, including BRCA reversion mutations, restoration of replication fork stability, and upregulation of drug efflux pumps are considered the main mechanisms of resistance [12,17,18]. Actually, the OReO trial on maintenance olaparib rechallenge in platinum-sensitive relapsed cancers found that around half of the patients did not benefit from a second PARP-i treatment [19]. Several agents have been attempted to overcome the PARP-i resistance so far. Multidrug resistance protein 1 is known as the efflux of chemotherapeutic drug and affect effectiveness and the accumulation of olaparib. Thus, this resistance could be improved by the administration of a p-glycoprotein inhibitor [10]. Epigenetic modification also affects the sensitivity to PARP-i. Since the methylation of BRCA1 or RAD51C genes leads to HRR defects, a demethylating agent might ameliorate PARP-i sensitivity [10]. CCAAT/enhancer binding protein β (C/EBPβ) is one of transcription factors in the HR pathway. C/EBPβ upregulates some HR genes including BRCA1, RAD51D, BRIT1, and BRIP1, and, consequently, induces the recovery of HR ability. Therefore, the inhibition or deletion of C/EBPβ could be a novel strategy to overcome the resistance [20]. However, although a clinical trial using bromodomain and extraterminal domain inhibitors that could reduce HR has been launched [21], reversing PARP-i resistance remains a tremendous challenge.

Recurrent ovarian, fallopian tube, or primary peritoneal cancers with a platinum-free interval (PFI) of more than 6 months have been defined as platinum-sensitive recurrences [7,19,22]; however, some evidence suggests that resistance to PARP-is may reduce responses to subsequent platinum-based chemotherapy, not only in ovarian cancer, but also in other malignancies [14,23]. A previous report showed that a duration of olaparib treatment ≥12 months could predict the response to subsequent platinum-based chemotherapy [13]. However, the efficacy of chemotherapy on disease progression after PARP-i was lower than expected according to the PFI [24]. Considering the biochemical mechanism of the antitumor effects of PARP-is, the results indicate that cancers that relapse during PARP-i therapy could have acquired platinum resistance. In the present study, although the enrolled patients were considered to have platinum-sensitive cancer, the PFS of the subsequent therapy in the CT group was limited to 3.1 months. The PFS almost coincides with that of previous clinical trials on platinum-resistant ovarian cancer [25,26]. The subgroup analyses of PFS in prognostically relevant patients demonstrated that age, pathological type, and prior line of chemotherapy were not independent prognostic factors. In addition, contrary to previous reports, the duration of olaparib use was not a significant predictive factor in this study. There was no significant difference between the two groups, although PARP-i dose reduction tended to result in a worse prognosis.

We then focused on bevacizumab use in subsequent therapy because the efficacy of bevacizumab in ovarian, fallopian tube, and primary peritoneal cancers has been established not only in the initial therapy, but also in recurrent settings with and without prior bevacizumab administration [2,3,27,28,29,30]. Statistically significant differences in PFS were confirmed between the CT and in CT + Bev groups. Additionally, the prolonged duration of PFS corresponded to the results of previous clinical trials. In other words, the present study demonstrated the efficacy of bevacizumab in patients with ovarian, fallopian tube, or primary peritoneal cancers who relapsed more than 6 months after olaparib maintenance therapy. The fact that only three patients received prior bevacizumab therapy in the present study could affect the clinical benefits experienced by these patients. Bevacizumab has been found to prolong progression-free survival not only in the first line [2,3], but also in the second-line treatment for platinum-sensitive ovarian cancer patients who had not received prior treatment with bevacizumab [28,29,30,31]. Moreover, the MITO-16B trial revealed that the efficacy of bevacizumab was found in the second-line therapy for patients with recurrent ovarian cancer who received the first-line bevacizumab [27]. These results support the result of the present study regardless of prior bevacizumab use.

The best strategy for patients with tumor relapse during PARP-i maintenance therapy remains unknown. A recent study demonstrated the efficacy and safety of bevacizumab-containing platinum-based chemotherapy in patients with recurrent ovarian, fallopian tube, or primary peritoneal cancers during PARP-i therapy [32]. However, these results did not demonstrate superiority over platinum-based chemotherapy without bevacizumab. In the present study, eight patients (44.4%) had not received a bevacizumab-containing regimen in their subsequent therapy. The main reasons for this were that a clinical trial on maintenance olaparib rechallenge was underway during the investigation period, and its efficacy was highly expected [19,33]. Additionally, bevacizumab-contained platinum-based chemotherapies followed by PARP-i maintenance therapy were not acceptable in previous clinical trials [7,8,22].

In terms of maintenance therapy, the OReO trial subsequently demonstrated the efficacy of PARP-i rechallenge in patients with recurrent ovarian, fallopian tube, or primary peritoneal cancers. However, the duration of prolonged PFS was only 1.5 and 2.5 months in patients with BRCA and non-BRCA mutations, respectively [19]. From the above, PARP-i rechallenge is unlikely to be a clinical option for patients who have already been treated with a PARP-i, while platinum-based chemotherapy with bevacizumab is a favorable clinical option for these patients. A maintenance therapy of bevacizumab combined with PARP-i might be considered as a clinical option in this setting. However, PAOLA-1 demonstrated the clinical benefits of maintenance therapy with olaparib and bevacizumab in patients with newly diagnosed advanced ovarian cancer who were receiving chemotherapy plus bevacizumab [6]. A NIRVANA-1 trial also evaluated the combination maintenance therapy with niraparib and bevacizumab in patients with newly diagnosed advanced ovarian cancer [34]. Although an AVANOVA2 trial revealed the efficacy of the combination maintenance therapy with niraparib and bevacizumab in a recurrent setting, no one received prior PARP-i treatment [35]. A NIRANOVA-R trial, which evaluated the combination maintenance therapy with niraparib and bevacizumab in a recurrent setting and enrolled patients who received prior treatment with a PARP-i, is underway [33]. Thus, there is no evidence using the combination maintenance therapy with bevacizumab and PARP-i in patients with platinum-sensitive recurrent ovarian cancer so far.

Adverse events were observed more frequently in the CT + Bev group than in the CT group. Although there were no statistically significant differences between the two groups, grade 2–4 hypertension occurred only after bevacizumab administration. Fortunately, no treatment was discontinued due to adverse events. On the other hand, treatment-related myeloid neoplasms, acute myeloid leukemia (AML), and myelodysplastic syndrome (MDS) are serious adverse effects considered as potentially PARP inhibitor-related [36]. The recent study demonstrated that PARP-is significantly increased the risk of AML and MDS when compared with the placebo treatment (odds ratio 2.63 [95% CI 1.13–6.14], *p* = 0.026). In fact, the incidence of AML and MDS in the olaparib group and in the placebo group was 1.5% and 0.8% in SOLO1 [5], 1.1% and 0.4% in PAOLA-1 [6], 0.3% and 0.0% in PRIMA [1], and 0.4% and 0.0% in ATHENA–MONO [37], respectively. On the other hand, SOLO2 trial, which is the first phase 3 trial of maintenance olaparib in patients with platinum-sensitive recurrent ovarian cancer, showed that 8% of patients were diagnosed with AML and MDS compared with 4% of patients in the placebo group [22]. Although no one was diagnosed with AML or MDS in the present study, these results could indicate that the incidence of AML and MDS in patients with platinum-sensitive recurrent ovarian cancer is higher than the incidence in patients with newly diagnosed ovarian cancer.

The present study has several limitations. First, it is difficult to draw unambiguous conclusions from the results because of the small number of patients. Second, HR and BRCA status could not be evaluated in the present study because the Japanese health insurance did not cover these tests for all enrolled patients. On the other hand, HR and BRCA status are known as biomarkers to predict the response to platinum-based chemotherapies. Thus, this missing information could affect the clinical outcomes in the present study.

## 5. Conclusions

We demonstrated the effect of PARP-i therapy on subsequent platinum-based chemotherapy in patients with recurrent ovarian, fallopian tube, or primary peritoneal cancers. The addition of bevacizumab to platinum-based chemotherapy significantly improved PFS in patients with relapsed tumors during PARP-i maintenance therapy. These results suggest that bevacizumab should be administered even to patients with platinum-sensitive cancer in a post-PARP-i setting. Nonetheless, further studies using a larger sample size are required to confirm our results.

## Figures and Tables

**Table 1:** Baseline patient characteristics (n = 18).

		Median (Range) or No. (%)
Age (years)		67 (30–80)
Follow-up period (month)		14.5 (1.4–35.0)
Performance status	0	15 (83.3)
	1	3 (16.7)
Primary site	ovary	12 (66.7)
	fallopian tube	1 (5.6)
	peritoneum	5 (27.8)
Histopathological type	serous	11 (61.1)
	endometrioid	4 (22.2)
	carcinosarcoma	1 (5.6)
	poorly differentiated	1 (5.6)
	other	1 (5.6)
FIGO stage *	I, II	4 (22.2)
	III, IV	14 (77.8)
Type of PARP-i	olaparib	15 (83.3)
	niraparib	3 (16.7)
Duration of PARP-i (months)		9.3 (6.4–18.6)
	6–12 months	16 (88.9)
	12 months	2 (11.1)
Dose reduction of PARP-i	yes	5 (27.8)
	no	13 (72.2)
Previous lines of chemotherapy	1	3 (16.7)
	2	10 (55.5)
	≥3	5 (27.8)
Prior bevacizumab use	yes	3 (16.7)
	no	15 (83.3)
Germline or somatic BRCA mutation	positive	1 (5.6)
	negative	5 (27.8)
	unknown	12 (66.6)

*: FIGO 2014.

**Table 2:** Univariable analyses of progression-free survival (n = 16).

		PFS (Months)		*p* Value
Age (years)				
	<67 vs. ≤67	7.2 ± 4.6	6.6 ± 3.8	0.681
Pathological type				
	serous vs. others	7.3 ± 3.9	5.2 ± 4.5	0.713
Duration of PARP-i (months)				
	<9 vs. ≤9	6.3 ± 3.5	7.4 ± 4.7	0.510
Previous lines of chemotherapy				
	≤2 vs. <2	5.9 ± 3.5	8.9 ± 4.7	0.267
Dose reduction of PARP-i				
	yes vs. no	5.7 ± 2.8	7.4 ± 4.5	0.320
Bevacizumab use in the subsequent therapy				
	yes vs. no	4.4 ± 3.4	8.8 ± 3.5	0.016

mean ± standard deviation.

**Table 3:** Regimen and efficacy of subsequent therapy (n = 18).

Regimen	n (%)	CRR (PR + CR)
Paclitaxel + Carboplatin	1 (5.6)	0 (0)
Paclitaxel + Nedaplatin	2 (11.1)	0 (0)
PLD + Carboplatin	5 (27.8)	1 (20)
Paclitaxel + Carboplatin + Bev	1 (5.6)	1 (100)
Gemcitabine + Carboplatin + Bev	2 (11.1)	2 (100)
PLD + Carboplatin + Bev	7 (38.9)	5 (71.4)

Abbreviations: CRR: clinical response rate; CR: complete response; PR: partial response; PLD: pegylated liposomal doxorubicin; Bev: bevacizumab.

**Table 4:** Grade 2–4 toxicity.

Events	CT (n = 8)			CT + Bev (n = 10)		
	G2	G3	G4	G2	G3	G4
Granulocytopenia	2 (25.0)	2 (25.0)	1 (12.5)	1 (10.0)	4 (40.0)	3 (30.0)
Anemia	2 (25.0)	2 (25.0)	1 (12.5)	5 (50.0)	3 (30.0)	0
Thrombocytopenia	2 (25.0)	1 (12.5)	0	1 (10.0)	1 (10.0)	4 (40.0)
Creatinine increased	1 (12.5)	0	0	1 (10.0)	1 (10.0)	0
Proteinuria	1 (12.5)	0	0	1 (10.0)	1 (10.0)	0
Vomit	1 (12.5)	0	0	1 (10.0)	0	0
Fatigue	1 (12.5)	0	0	1 (10.0)	1 (10.0)	0
Perforation/Fistula	0	0	0	0	0	0
Peripheral neuropathy	1 (12.5)	0	0	1 (10.0)	0	0
Hand-foot syndrome	0	0	0	0	0	0
Hypertension	0	0	0	2 (20.0)	1 (10.0)	0
Bleeding	0	0	0	0	0	0
Thromboembolic event	0	0	0	0	0	0
Cardiac disorders	0	0	0	0	0	0
Hypersensitivity	0	0	0	0	1 (10.0)	0

## Data Availability

The data generated or analyzed during this study are available from the corresponding author on reasonable request. The data are not publicly available due to them containing information that could compromise participants’ privacy.
